# Comprehensive review of bacterial death mechanism on nanopillared nanostructured surfaces

**DOI:** 10.1007/s12551-025-01319-5

**Published:** 2025-05-20

**Authors:** Dimuthu Wijethunge, Asha Mathew, Prasad K. D. V. Yarlagadda

**Affiliations:** School of Engineering, University of Southern Queensland, 37 Sinnathamby Blvd, Springfield Central QLD, 4300 Australia

**Keywords:** Nanostructured surfaces, Bactericidal surfaces, Antibacterial surfaces, Mechanobactericidal effect

## Abstract

Antibiotic resistance presents a significant global concern, worsened by overuse and limited development of new antibiotics. Medical implants, in particular, are increasingly susceptible to bacterial infections. To prevent biofilm formation on implants, it is essential to design specialized surface characteristics that either kill bacteria or inhibit their growth. Nanostructures resembling those found in nature, such as cicada wings, exhibit pronounced antibacterial efficacy. Drawing inspiration from these natural surfaces, artificial nanostructures made with similar features have demonstrated bactericidal effect. The bactericidal mechanism in nanostructures may seem simple, as the nanofeatures pierce through bacterial cells, leading to their death. However, research has shown that it is more complex and requires thorough investigation. Several studies indicate that while the bactericidal mechanism is initiated by mechanical contact, the precise killing process remains uncertain. Numerous experimental and theoretical investigations have aimed to elucidate the exact killing mechanism, yielding diverse conclusions and hypotheses, including cell death attributed to creep failure, motion-induced shear failure, apoptosis-induced programmed cell death and autolytic cell death, among others. This study undertakes a comprehensive review of all proposed death mechanisms. Moreover, it draws conclusions on the killing mechanism by meticulously analyzing the properties of bacterial membranes, their mechanosensing and adhesion mechanisms, energy-based models for bacterial adhesion, and experimental outcomes regarding the bactericidal efficacy of surfaces exhibiting diverse geometries.

**Graphical Abstract:**

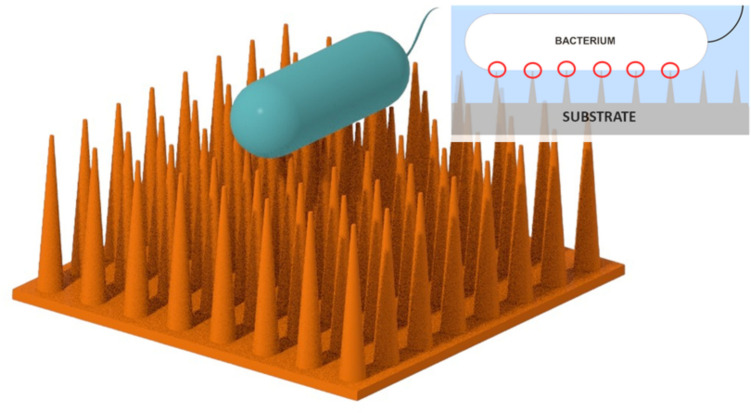

## Introduction

Antibiotic resistance has created significant health concerns as it diminishes the effectiveness of current medications against bacterial infections (Akram et al. 2023). The overuse of antibiotics has created an excellent background for bacteria to evolve, developing resistance. Additionally, the limited development of new antibiotics attributed to economic, technological, and regulatory challenges faced by pharmaceutical companies, further aggravates the crisis. Among various forms of bacterial infections, those affecting medical implants are becoming significantly more prevalent, leading to implant failures (Zimmerli et al. 2004) and causing serious health issues, even death (Steckelberg and Osmon 2000). Moreover, the emergence of antibiotic resistance could increase mortality in patients with implant infections. Implant surfaces, as they are designed to facilitate tissue regeneration, are prone to biofilm formation. Preventing biofilm formation requires strategies to either prevent bacteria from adhering to the implant surface or eliminate the bacteria adhered to the surface. Physicochemical surface modifications targeted for cell and protein repellence can reduce bacterial attachment. However, this approach may not be suitable for implant surfaces, which require the facilitation of tissue regeneration. Therefore, implant surfaces should support cell attachment, even though it creates the adverse effect of increased bacterial attachment (Bacakova et al. 2011). This necessitates the introduction of special surface features to implant surfaces, which can either kill the bacteria attached to the surface or inhibit bacterial growth.

Bactericidal surfaces are not uncommon, and various surfaces employ different mechanisms to eliminate bacteria from their surfaces. Some surfaces chemically react with bacteria, leading to bacterial death. These surfaces contain substances, such as nanoparticles, antibiotics, or ions, that interact with bacteria (Bakhshandeh et al. 2017; Wang et al. 2017; Amin Yavari et al. 2016; Necula et al. 2012). However, over time, the effectiveness of these interactions will diminish. Additionally, some of those substances can also be cytotoxic to other human cells (Singh and Ramarao 2012). Special types of bactericidal surfaces with nanopillars in their surface have shown excellent bactericidal properties (Ivanova et al. 2012). These types of bactericidal surfaces offer numerous advantages as the bactericidal effect is long-lasting due to the durability of the nanopillars. They are effective against a variety of cells, reduce the chance of antimicrobial resistance development, and can be combined with other antibacterial modes through coating. Bacterial death on such surfaces initiates upon mechanical contact, although the precise mechanism remains incompletely understood. This bactericidal effect is also known as mechanobactericidal effect. It is conclusive that mechanical contact, which can apply considerable stress on the bacterial cell wall and outer membrane, is a prerequisite. This is supported by the observation of significant bacterial death occurring only on surfaces with nanofeatures, in contrast to those without such features, even when surface material is made of the same material (Hasan et al. 2017; Ivanova et al. 2013). Whether bacterial death is caused solely by mechanical cell rupture or involves other mechanisms is still uncertain. There are several hypotheses, such as cell death caused by the nanostructure piercing the cell (Jenkins et al. 2020), creep failure, (Liu et al. 2019) motion-induced shear failure, (Bandara et al. 2017) oxidative stress-induced cell death, (Jenkins et al. 2020) and a combination of oxidative stress-induced cell death and apoptosis-induced death (Zhao et al. 2022). Even though some of these hypotheses are partially backed by experiments to some extent, the conclusions drawn from these experiments were not strong enough to fully elucidate the actual bacterial killing mechanism in nanostructures.

In this review, we have comprehensively analyzed the existing research to draw solid conclusions about bacterial killing mechanisms in nanostructures. In the process, we have explored various aspects, including mechanosensing in bacteria, bacterial attachment mechanisms to surfaces, energy-based models, influence of geometrical parameters, and finally, oxidative stress-related bacterial death. Our main aim is to construct a clear and comprehensive understanding of the actual bactericidal phenomenon.

## Analysis

### Cell composition and mechanical properties of cell membranes

**Fig. 1:**
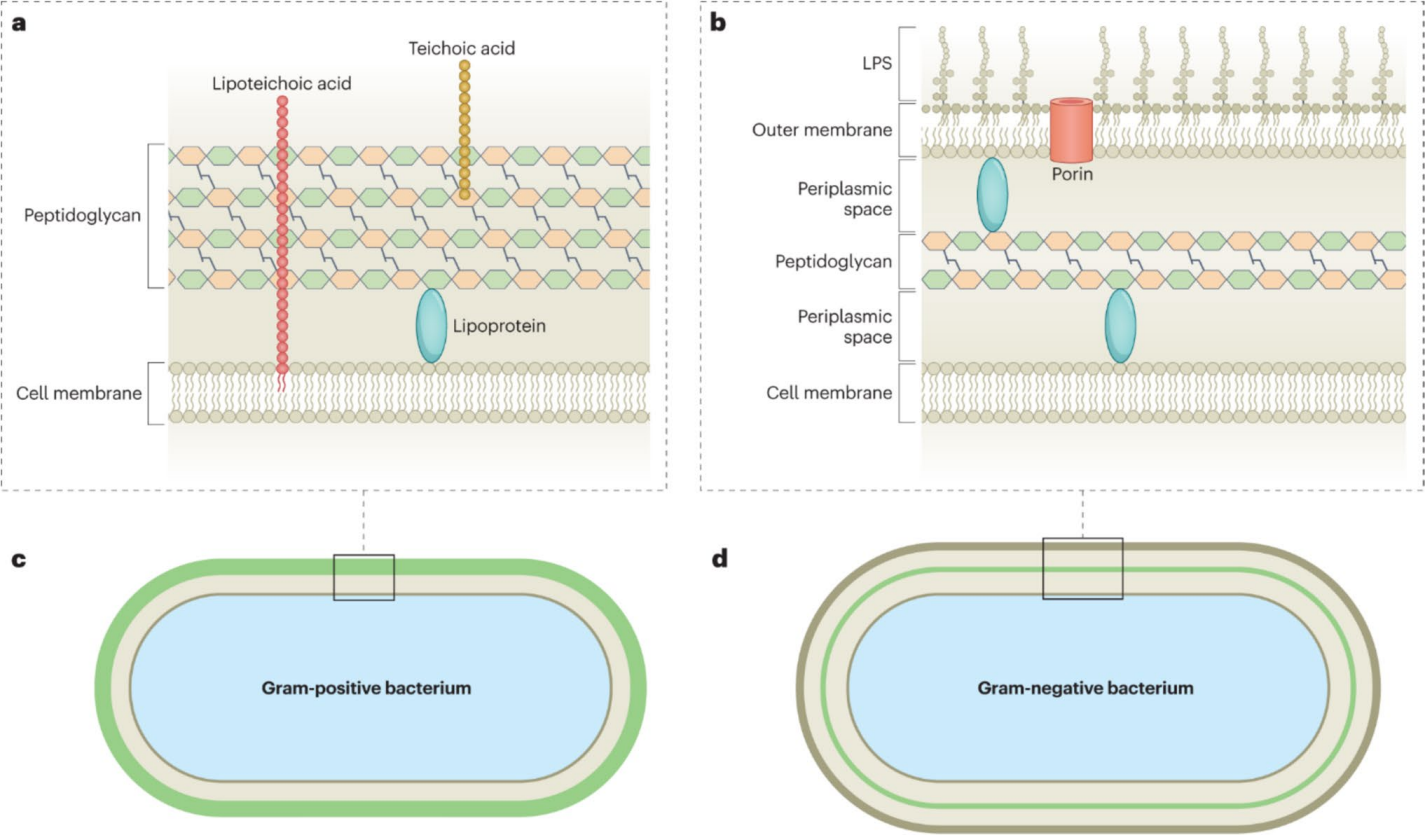
Representations of composition and structure of bacterial membranes: **a** Gram-positive bacterium and **b** Gram-negative bacterium, shown as cross-sections through the cell envelope. Cross-sectional representations of the whole cell: **c** Gram-positive bacterium and **d** Gram-negative bacterium. The cytoplasm is shown in light blue. The inner or cytoplasmic membrane, composed of phospholipids and inner membrane proteins, surrounds the cytoplasm. The cell wall, indicated by a green line, predominantly consists of peptidoglycan. Periplasm is known as the overall space between inner and outer membrane, approximately 15 nm wide in Gram-negative bacteria. The outer membrane, an integral component of Gram-negative bacteria, is composed of phospholipids, lipopolysaccharides, and outer membrane proteins (reprinted with permission from ref (Sun et al. 2022) Copyright 2023 Nature Publication)

Before diving into the analysis of mechanobactericidal effects, it is important to establish a solid understanding of bacterial cell composition and structure. This foundational knowledge is necessary to accurately define the factors contributing to bacterial death and also to comprehend some of the predicted bacterial death mechanisms, such as oxidative stress-induced cell death. Figure 1 illustrates the composition of Gram-negative bacteria. Cytoplasm is a vital component of bacterial cells, comprising water, enzymes, and various cellular structures such as ribosomes, chromosomes, and plasmids. In contrast to eukaryotic cells, bacterial genetic material is not enclosed within a distinct nucleus but is dispersed throughout the cytoplasm. The chromosome in bacteria carries the genetic instructions essential for bacterial replication. The inner or cytoplasmic membrane, which encloses the cytoplasm, functions as a protective barrier that regulates the influx and efflux of materials while selectively interacting with the extracellular environment. Inner membrane consist of phospholipids and inner membrane proteins (Ruiz et al. 2008). Following the inner membrane, bacterial cells are enveloped by the cell wall, a rigid structure primarily consisting of peptidoglycan (Weidel et al. 1960; Weidel and Pelzer 1964). The strength of the wall keeps the cell intact, preventing cell from bursting when there are large pressure differences between the cytoplasm and the environment. Periplasm is known as the overall space between the inner and outer membrane. Gram-negative bacterial cells have a single-layered cell wall, typically ranging from 1 to 7 nm in thickness, whereas Gram-positive bacterial cells exhibit a thicker cell wall, approximately 20–100 nm, reinforced by multiple cross-linked layers (Cabeen and Jacobs-Wagner 2005). Even though the cell wall of Gram-negative bacteria is comparatively weaker, it is further strengthened by an outer membrane composed of phospholipids in the inner leaflet, lipopolysaccharide (LPS) in the outer leaflet, and outer membrane protein (OMP)s (Silhavy et al. 2010). Typically, LPS comprise O-antigens, core polysaccharide and lipid (Silhavy et al. 2010; Rojas et al. 2018). Notably, the outer membrane possesses an overall negative charge, contributing to its functional properties and interactions with the extracellular environment.

For our investigation of the mechanobactericidal effect, the mechanical properties of bacterial cells are of primary interest. Early research indicates that the cell wall of bacterial cells exhibits elasticity and flexibility, (Marquis 1968; Yao et al. 1999; Doyle and Marquis 1994) as evidenced by the observation of expansion and contraction in isolated sacculi, a phenomenon subsequently corroborated by multiple studies. The measurement of the cell wall elasticity in *Escherichia coli* (*E. coli*) bacteria revealed an approximate 300% increase from its relaxed state, providing quantitative insight into the extent of elasticity inherent in these bacterial cell walls (Yao et al. 1999).

The cell wall plays a crucial role in maintaining the mechanical integrity of the cell, withstanding various mechanical stresses including turgor pressure, (Höltje 1998; Koch 1988) which originates from the pressure exerted by cytoplasmic fluid against the cell wall. The mechanical robustness of the cell wall is critical in preserving cellular integrity amid fluctuations in pressure (Deng et al. 2011). Rojas et al. have demonstrated the substantial contribution of the outer membrane to maintaining cell integrity (Rojas et al. 2018). Any damage to the outer membrane a compromised coupling between outer membrane and cell wall can lead to significant cell deformation and reduced survival rates in response to mechanical shocks and stresses (Rojas et al. 2018).

### Mechanism of bacteria adhesion to the surface

Bacteria naturally exhibit a preference for surface attachment and the formation of biofilms, which are community of bacteria embedded in a self-produced extracellular matrix. In fact, the majority of bacteria in diverse environments thrive within biofilms, signifying their evolutionary adeptness at adhering to surfaces and adopting an immobile lifestyle within these structures. However, some cells are not entirely immobile, as they differentiate into swarmer cells which are elongated and hyperflagellated forms that facilitate surface movement, enabling rapid expansion of the colony. This behaviour is also evident in motile strains, which exhibit faster colony expansion compared to non-motile counterparts (Díaz et al. 2009).

**Fig. 2:**
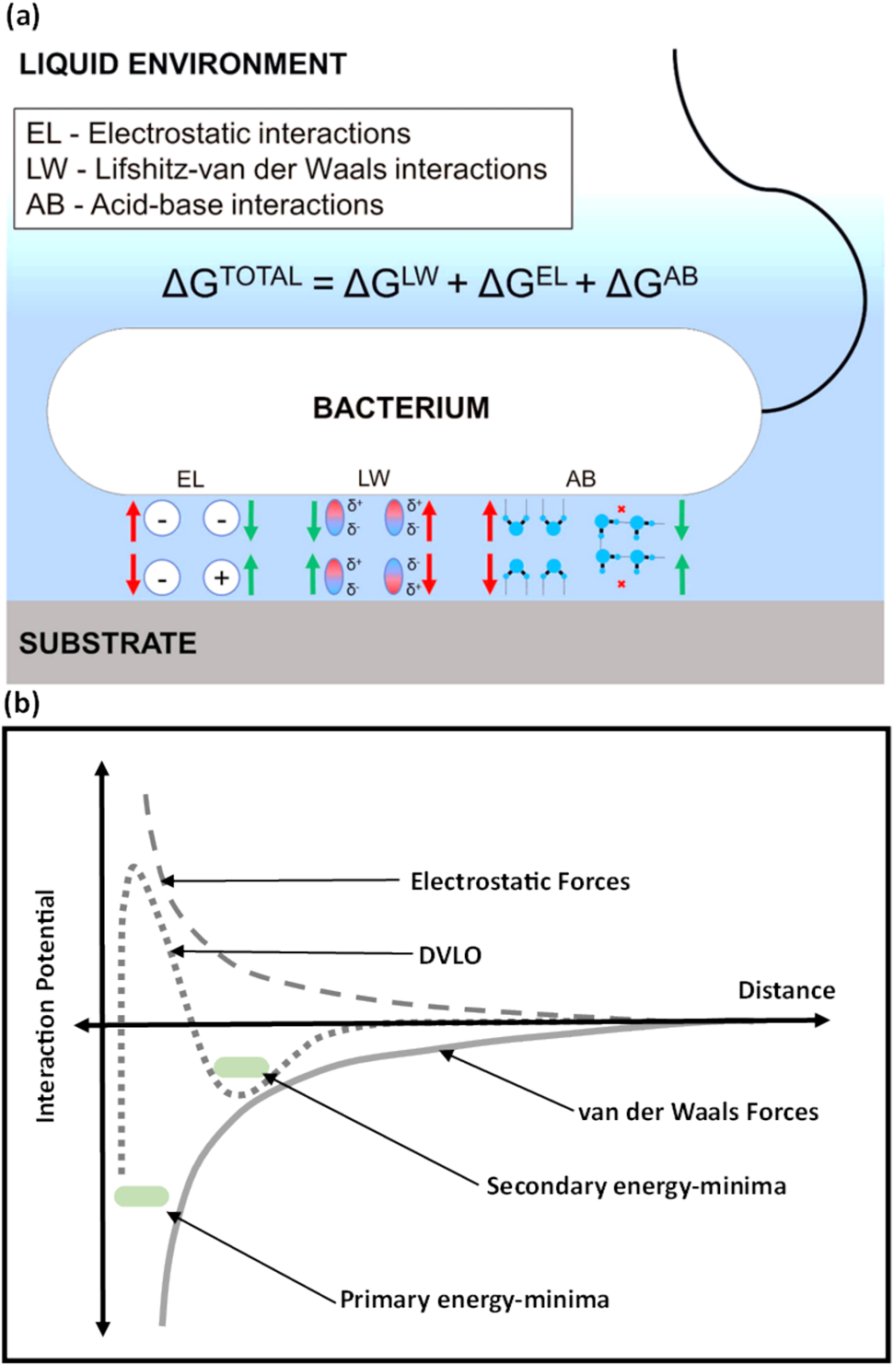
**a** Illustration of the electrostatic double layer, Lifshitz-van der Waals, and acid–base interactions which occur between bacteria and surfaces. Reprinted with permission from ref (Zheng et al. 2021) Copyright 2021 Frontiers Publication. **b** A graph illustrating the Derjaguin Landau Verwey Overbeek (DLVO) interaction energy as a function of distance, depicting both secondary and primary energy minima

The process of bacterial adhesion to a surface unfolds in several sequential steps. Initially, bacteria navigate towards the surface in a fluid medium. Motile bacteria can actively move toward the surface, with the assistance of their appendages and sensory mechanisms (Schweinitzer and Josenhans 2010; Pratt and Kolter 1998). In contrast, non-motile bacteria solely rely on Brownian motion and interatomic forces to reach close proximity to a surface (Berne et al. 2018). Upon proximity to the surface, adherence is primarily governed by several forces according to the extended Derjaguin − Landau − Verwey − Overbeek (XDLVO) theory (Harimawan et al. 2013). These include attractive van der Waals forces with a relatively long range of up to 1 μm, repulsive electrostatic forces, and acid–base interaction forces that can be either repulsive or attractive depending on the specific interactions between the bacterial surface and the substrate surface (Berne et al. 2018; Costerton et al. 1995; Ren et al. 2018; Camesano and Logan 2000). As shown in Fig. 2, DVLO theory elucidates two energy minima: secondary shallow energy-minimized position and a primary deep energy-minimized position, separated by a significant energy barrier. Despite the insights provided by the DLVO theory, steric interactions, particularly at shorter ranges, exert a dominant influence on adhesion dynamics, contributing to an energy barrier potentially greater than predicted (Camesano and Logan 2000). Furthermore, Lorenzetti et al. highlighted that DLVO theory is effective when the free energy of the binding process is negative, such as with electrostatic attraction (Lorenzetti et al. 2015). However, for cases where both surfaces are negatively charged, such as negatively charged titanium surfaces and negatively charged bacterial surfaces, DLVO theory is not effective.

Bacteria initially adhere to a surface in a reversible manner, positioning themselves at a secondary energy-minimized state (Carniello et al. 2018). Subsequently, they employ their appendages to effectively overcome the potential energy barrier and transition to the primary energy-minimized position. Despite the strong attachment, these appendages act as nano springs, still allowing for reversibility in adhesion (Westen et al. 2018). Certain bacterial species utilize flagella or other thin filamentous protein to enhance adhesion to the surface (Pratt and Kolter 1998; Palmer et al. 2007). After the initial reversible attachment, the bond strength between bacteria and the surface intensifies as atoms reposition themselves. Interfacial water is displaced owing to the bacteria’s hydrophobic nature. Additional surface contacts are created by bacterial appendages such as pili or fimbriae, further enhancing the irreversibility of attachment (DeBenedictis et al. 2016). This attachment is reinforced by the secretion of Extracellular Polymeric Substance (EPS) and other molecular adhesins, which contain numerous proteins facilitating robust surface attachments (Carniello et al. 2018). Cell repositioning to strengthen interactions with the surface is observed for some bacterial cells (Petrova and Sauer 2012). As cells adhere to the surface, deformation occurs, subsequently increasing bonding as the atoms in the bacterial membrane approach the surface. Bacteria possess the remarkable ability to sense contact forces, including inward compressive forces during surface attachment and tensile forces upon detachment. This mechanosensing ability plays a pivotal role in strengthening surface adherence, involving sensory organelles such as flagella and pili, as well as stress-detecting proteins detecting membrane deformations (Straub et al. 2019). These proteins are activated by contact pressure, enabling bacteria to sense and respond to mechanical forces (Gordon and Wang 2019; Harapanahalli et al. 2015).

Collectively, these findings suggest that bacteria have evolved sophisticated mechanisms for surface attachment, where adhesion forces can reach considerable magnitudes and become irreversible. Bacteria employ multiple mechanisms to firmly attach to surfaces, which may result in significant cell deformations when adhering to nanostructures. Irreversible adhesion is primarily driven by the specific mechanisms of bacterial attachment. Bacteria possess sensory mechanisms and intrinsic intelligence in place to detect and avoid such stress, but further exploration is needed to fully understand bacterial response and adaptation to these mechanical challenges.

### Gaining insights from natural mechanobactericidal surfaces

The nanostructures found on certain insect wings and animal skins have gained significant attention due to their potential antibacterial properties. It was Initially believed that cicada wings were hydrophobic to prevent biofouling, (Sun et al. 2009) later discovered they were not efficient in repelling bacteria. Ivanova et al. conducted further studies, uncovering that bacteria cells (Pseudomonas aeruginosa) adhered to the surface of cicada wings. Remarkably, the nanopatterns present on the wings seemed capable of penetrating the cell surface, leading to the death of bacteria (Ivanova et al. 2012). It was observed that cicada wings coated with 10 nm gold exhibited bactericidal properties, despite a decrease in hydrophobicity. This research shed light on how nanostructures can kill bacteria through mechanical activity, with the bactericidal effect primarily driven by the topological properties of the nanopattern rather than surface chemistry. Hasan et al. investigated the contact-based death of various Gram-positive and Gram-negative bacteria using cicada wings. The findings revealed that the Gram-negative bacteria exhibited susceptibility, leading to death, while Gram-positive bacteria demonstrated resistance to the effect (Hasan et al. 2013). Some studies have shown that several varieties of cicada wings are capable of killing not only prokaryotic microbes but also eukaryotic microorganisms, such as *Saccharomyces cerevisiae* (Nowlin et al. 2015).

**Fig. 3:**
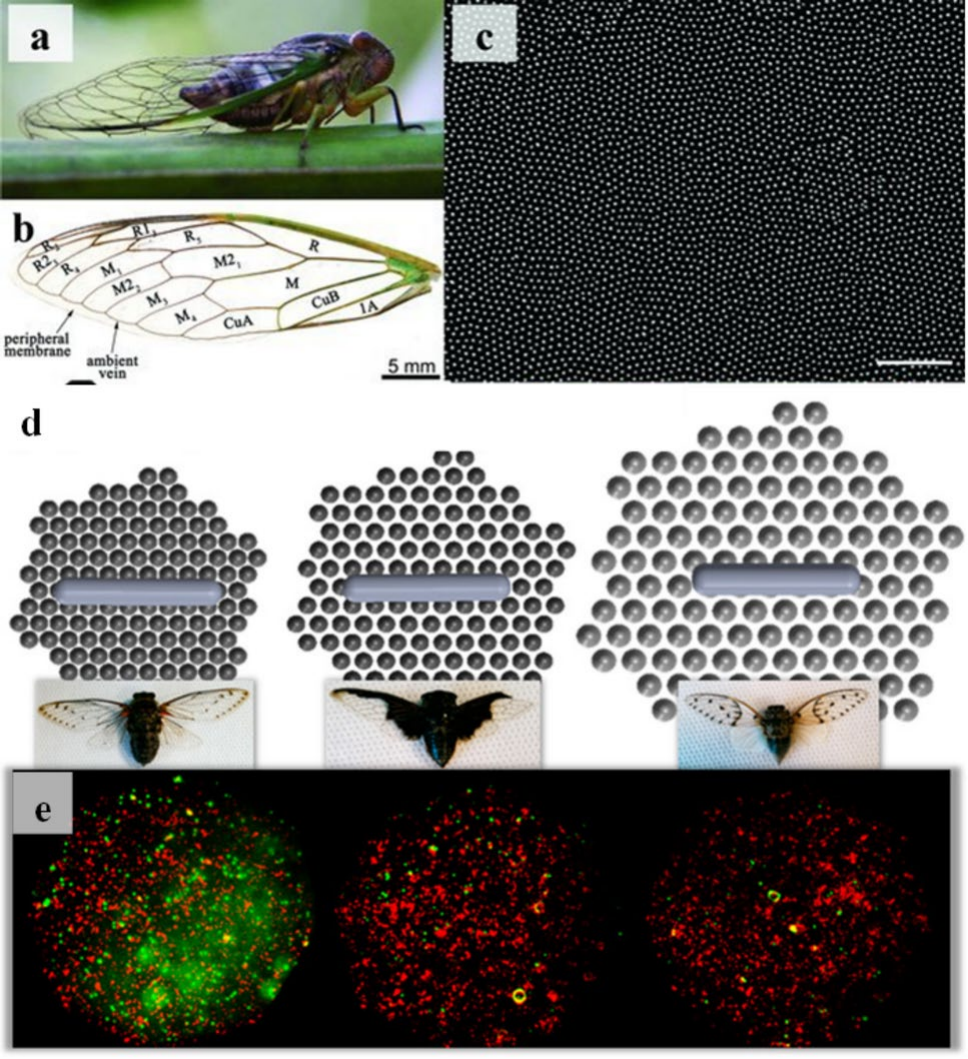
**a** Photograph of cicada, *Psaltoda claripennis*. **b** Illustration depicting the structural physiology of the forewing of *Psaltoda claripennis*, highlighting the major veins. **c** Scanning electron micrograph (SEM) captured at 25,000 × magnification revealing nanopatterns in upper surface of the forewing. The surface showcases an array of nanoscale pillars arranged with approximately hexagonal spacing. A scale bar of 2 μm is provided for reference (reprinted with permission from ref (Ivanova et al. 2012) Copyright 2012 Wiley Publication). **d** Images illustrating samples of various cicada species with different nano patterned wings, arranged from right to left: M. intermedia, *C. aguila* and *A. spectabile*. **e** Fluorescent micrographs showing dead/injured Pseudomonas fluorescens cells in green and undamaged Pseudomonas fluorescens cells in red (reprinted with permission from ref (Kelleher et al. 2016) Copyright 2016 ACS Publication)

Through meticulous examination, they concluded that factors such as downward adhesion force and its rate of increase upon surface contact are crucial for enhancing bactericidal efficiency, affecting both Gram-positive and Gram-negative bacteria. Another study utilizing various cicada wings suggested that even minor changes in nanopattern topology can significantly impact bactericidal effectiveness. Notably, one wing type with larger nanopillar diameter and spacing exhibited reduced bacterial death compared to others. Mainwaring et al. observed the bactericidal efficiency of three distinct dragonfly wing topologies (Mainwaring et al. 2016). Through meticulous examination of variations in topology among the dragonfly wings, they concluded that the factors such as downward adhesion force and its rate of increase upon surface contact are crucial for enhancing bactericidal efficiency, affecting both Gram-positive and Gram-negative bacteria. Another study utilizing various cicada wings suggested that even minor changes in nanopattern topology can significantly impact bactericidal effectiveness (see Fig. 3b) (Kelleher et al. 2016). In particular, one wing type with larger nanopillar diameter and spacing exhibited reduced bacterial death compared to others. Similar to cicada wings, gecko skin offers valuable insights into antibacterial nanostructured surfaces and contact-based bacterial death mechanisms. Watson et al. demonstrated that gecko skin, which has nanostructures similar to those found on cicada wings but with double the spacing, can eliminate bacteria using a similar mechanism (Watson et al. 2015). Later Li et al. successfully replicated gecko skin patterns on acrylic surface, retaining antibacterial properties (Li et al. 2016). Truong et al. conducted an analysis of the susceptibility of Staphylococcus aureus and Pseudomonas aeruginosa bacteria based on their physiological maturity on damselfly surfaces (Truong et al. 2017). Their study revealed a higher tendency for attachment in physiologically young S. aureus and mature P. aeruginosa bacterial cells compared to other growth stages.

These research findings have demonstrated the highly effective bactericidal properties of nanostructured surfaces, including their ability to eliminate some eukaryotic microorganisms. The studies have revealed that bacterial death on nanostructured surfaces is primarily originates from physical contact rather than chemical reactions, emphasizing the critical role of topological properties in governing killing mechanisms. The observed increased resistance in Gram-positive bacteria highlights the significance of membrane thickness in contact-based bacterial death.

Inspired by naturally occurring bactericidal nanosurfaces, researchers have developed and investigated artificial nano surfaces with various topologies using different materials. These artificial surfaces aim to replicate or enhance the bactericidal properties observed in nature. A surface with high nanoprotrusions made with black silicon was one of the first reported artificial hydrophilic surfaces that exhibited a physical bacterial killing mechanism effective against both Gram-negative and Gram-positive bacteria (Ivanova et al. 2013). Later, nanostructured surfaces with similar bactericidal properties were fabricated using various materials such as black silicon (Hasan et al. 2013; Wang et al. 2016), titanium (Hasan et al. 2017; Lorenzetti et al. 2015; Bhadra et al. 2015), titanium oxide (TiO_2_) (Jaggessar et al. 2018), PMMA (Dickson et al. 2015), gold (Wu et al. 2016), diamond (Fisher et al. 2016), diamond-coated black silicon (May et al. 2016), TiO_2_-coated polystyrene (Salatto et al. 2023), graphite (Ivanova et al. 2017), stainless steel (Peter et al. 2020), and aluminium alloy (Hasan et al. 2020).

### Insights from nanostructured surface geometrical parameters

**Fig. 4:**
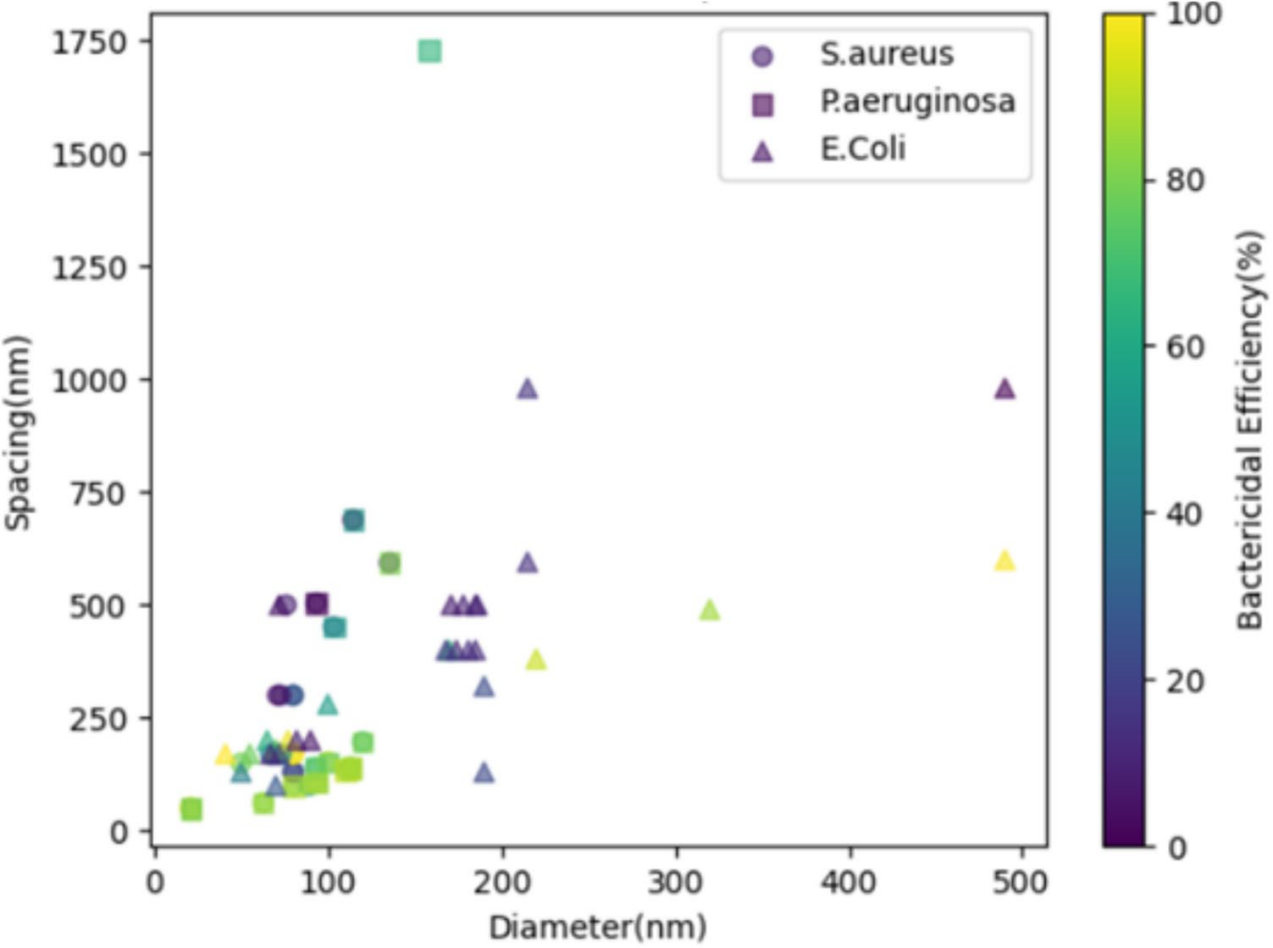
The plot illustrates bactericidal efficiency of nanopillar with different diameter and spacing for three bacterial strains: *S. aureus*, *P. aeruginosa*, and *E. coli*

Extensive research and reviews have focused on the geometrical influence on bactericidal effects in nanostructured surfaces, with the primary aim of optimizing nanofeatures to enhance their bactericidal effectiveness. In this work, our main focus is to identify key takeaways and gain insights from the empirical evidence presented by these variations, aiming to enhance our comprehension of bactericidal killing mechanisms. In nanostructured surfaces, key geometrical features include height, spacing or pitch, tip diameter, aspect ratio, and topography. Nanostructures can be classified based on their topography into distinct categories such as nanopillars, nanocolumns, nanocones, nanowires, nanospinules, and nanospikes. A comprehensive comparison of these features can be found in many research works (Modaresifar et al. 2019; Ganjian et al. 2019; Senevirathne et al. 2021; Ishantha Senevirathne et al. 2021). If stress and deformation are major contributing factors to surface bactericidal efficiency, then surface topography, pitch, and tip diameter become important factors, as they can greatly influence the stress and deformation. In this work, we have used data from numerous research studies on nanopillar type bactericidal surfaces to investigate the correlation between the pitch (spacing) and diameter of the surface pattern and bacterial killing efficiency. These data, available for nanopillar surfaces with varying pitch and tip diameter, spans different types of bacteria, including *Staphylococcus aureus*, *Pseudomonas aeruginosa*, and *Escherichia coli*, and is plotted in Fig. 4. The detailed data used for the plot are given in Table 1. Here we, focus only on a certain type of surface topology (nanopillar surfaces) to gain insights into the killing behaviour, rather than determining the optimum geometry. According to the data, it is evident that an increase in spacing and decrease in diameter correlate with a decrement in bactericidal efficiency across all three bacterial types. This can be attributed to the fact that increased spacing and diameter reduce the stress and deformation on the bacterial membrane, which have been correlated with bacterial efficiency. Several finite element-based studies have supported this correlation, providing clear illustrations of stress distribution in cell membranes (Velic et al. 2021a, 2019, 2021b). According to these studies, the highest stress occurs near the tip contact area of the membrane as it gets highly deformed (Velic et al. 2021a). Therefore, the reduction in tip diameter and the presence of sharpened topography can increase the stress on cell membranes.

**Table 1:** The killing efficiencies of nano pillared surfaces with different geometries (diameter and spacing) on three different bacteria types, namely *S. aureus*, *P. aeruginosa*, and *E. coli*

*S. aureus*			*P. aeruginosa*			*E. coli*		
Diameter	Spacing	Efficiency	Diameter	Spacing	Efficiency	Diameter	Spacing	Efficiency
93.3	503.7	12 (Linklater et al. 2019)	93.3	503.7	32 (Linklater et al. 2019)	72	500	4 (Dickson et al. 2015)
103.3	451.5	37 (Linklater et al. 2019)	103.3	451.5	68 (Linklater et al. 2019)	70	170	23.5 (Dickson et al. 2015)
114.5	688.3	4 (Linklater et al. 2019)	114.5	688.3	68 (Linklater et al. 2019)	190	320	19 (Dickson et al. 2015)
135.5	593.3	15 (Linklater et al. 2019)	135.5	593.3	87 (Linklater et al. 2019)	215	595	11.33 (Dickson et al. 2015)
62.7	62.1	85 (Linklater et al. 2017a)	158	1729	76 (Linklater et al. 2019)	50	130	35 (Michalska et al. 2018)
80.3	99.5	78 (Linklater et al. 2017a)	62.7	62.1	88.74 (Linklater et al. 2017a)	70	180	65 (Michalska et al. 2018)
92.7	139.7	75 (Linklater et al. 2017a)	80.3	99.5	92 (Linklater et al. 2017a)	100	280	45 (Michalska et al. 2018)
100	153	92.3 (Bhadra et al. 2018)	92.7	139.7	81 (Linklater et al. 2017a)	170	400	50 (Michalska et al. 2018)
110	135	78.7 (Bhadra et al. 2018)	100	153	83.7 (Bhadra et al. 2018)	220	380	75 (Michalska et al. 2018)
120	197	73.5 (Bhadra et al. 2018)	110	135	93.9 (Bhadra et al. 2018)	320	490	70 (Michalska et al. 2018)
80	130	31 (Wu et al. 2018)	120	197	85.9 (Bhadra et al. 2018)	490	600	81 (Michalska et al. 2018)
80	130	23 (Wu et al. 2018)	21	49.9	98.97 (Linklater et al. 2017b)	70	100	22 (Michalska et al. 2018)
80	170	100 (Wu et al. 2018)	93	106.6	99.79 (Linklater et al. 2017b)	190	130	18 (Michalska et al. 2018)
80	170	98 (Wu et al. 2018)	113	139.7	85.4 (Linklater et al. 2017b)	215	980	12 (Michalska et al. 2018)
80	300	26 (Wu et al. 2018)				55	170	63.25 (Lohmann et al. 2022)
80	300	31 (Wu et al. 2018)				77	200	79 (Lohmann et al. 2022)
89	100	62 (Modaresifar et al. 2020)				174	400	9 (Lohmann et al. 2022)
73	170	50.8 (Modaresifar et al. 2020)				178	500	7.5 (Lohmann et al. 2022)
67	170	47 (Modaresifar et al. 2020)				41	170	81.5 (Lohmann et al. 2022)
73	300	14 (Modaresifar et al. 2020)				65	200	51 (Lohmann et al. 2022)
71	300	8.78 (Modaresifar et al. 2020)				168	400	14 (Lohmann et al. 2022)
76	500	12.89 (Modaresifar et al. 2020)				171	500	6.3 (Lohmann et al. 2022)
21	49.9	97.8 (Linklater et al. 2017b)				72	170	17 (Lohmann et al. 2022)
93	106.6	95 (Linklater et al. 2017b)				82	200	7.7 (Lohmann et al. 2022)
113	139.7	89.617 (Linklater et al. 2017b)				181	400	12 (Lohmann et al. 2022)
50	150	80 (Izquierdo-Barba et al. 2015)				185	500	7.29 (Lohmann et al. 2022)
						67	170	8.7 (Lohmann et al. 2022)
						90	200	4.72 (Lohmann et al. 2022)
						185	400	9.7 (Lohmann et al. 2022)
						186	500	8.65 (Lohmann et al. 2022)

**Fig. 5:**
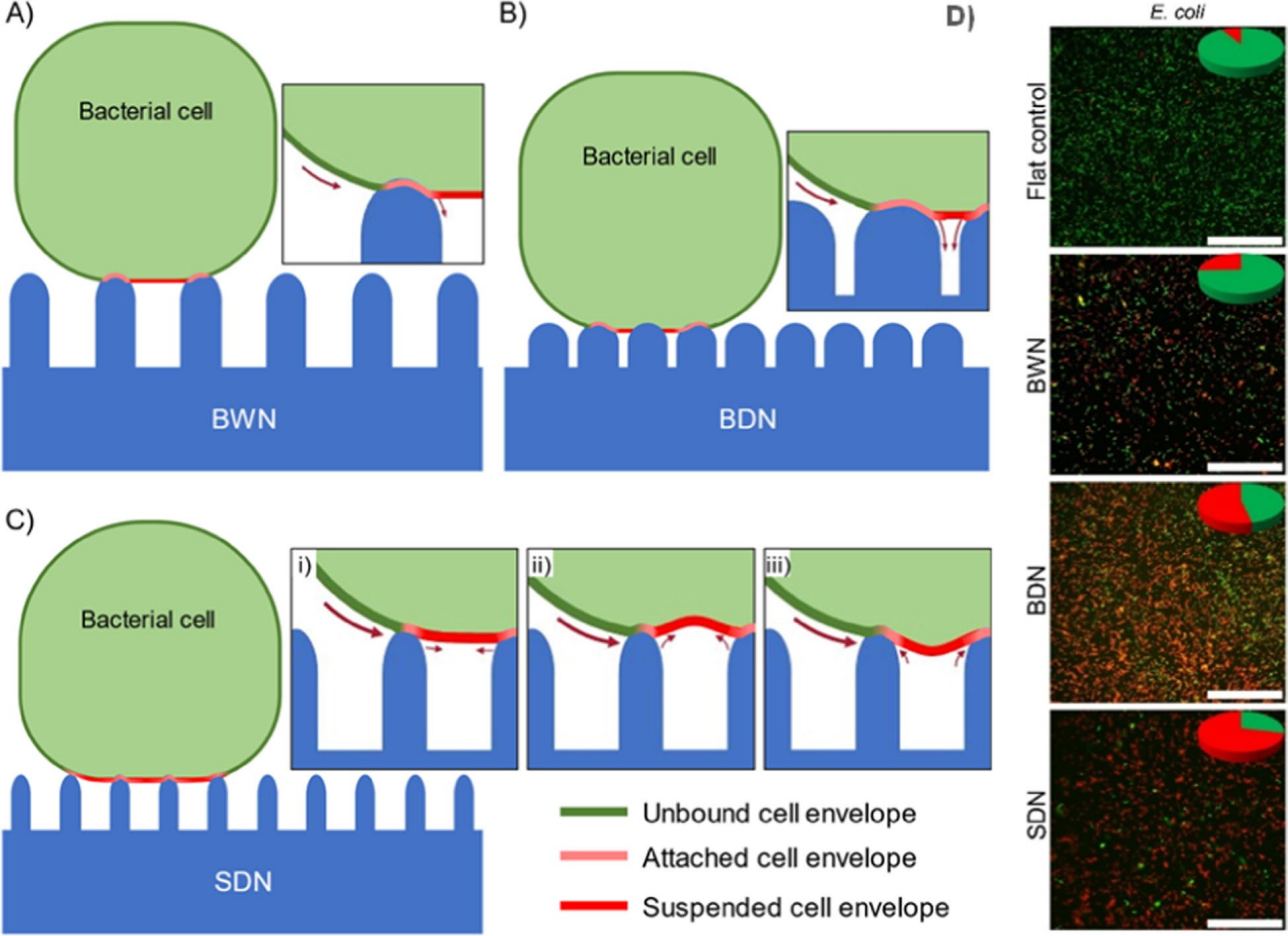
Illustration of bacterial attachment to Nano pillared surfaces and its impact on cell envelope stretching. Bound regions (red and pink) exhibit distinct stretching behaviours compared to unbound regions (green). On **A** blunt and wide nanopillars (BWN) and **B** blunt and dense nanopillars (BDN) surfaces, bacterial cells show stretched cell envelopes in unbound regions due to reduced intrinsic contact area compared to flat controls. Conversely, on sharp and dense nanopillars (SDN) surfaces (**C**), unbound regions stretch more due to smaller intrinsic contact area. FIB-SEM images reveal three possible deformations of suspended cell envelopes: flat (i), inward (ii), or outward (iii). **D** Fluorescence micrographs displaying bacteria (*E. coli*) adhered to BWN, BDN, and SDN nanopillared surfaces. LIVE/DEAD stain highlights viable (green) and damaged (red/orange) bacteria, with relative proportions depicted in inset pie charts. Scale bars denote 40 μm. Reprinted with permission from ref (Ishak et al. 2021) Copyright 2021 Elsevier Publication

In the investigation carried out by Michalska et al., they observed that pillars featuring sharpened tips exhibited bactericidal effects of approximately 80%, even when the spacing between them was approximately 600 nm (Michalska et al. 2018) Additionally, they suggest that bacterial death is more likely due to deformation-induced rupture with blunt tip shapes, while with sharpened tips, it occurs through penetration. A similar study conducted by Ishak et al. (Fig. 5) demonstrated that poly(ethylene terephthalate) surfaces with sharp dense pillars (40-nm tip diameter, 122-nm pitch) exhibited the highest bactericidal activity, followed by dense blunt pillars (70-nm tip diameter, 122-nm pitch), while the lowest activity was observed on blunt wide nanopillars (70-nm tip diameter and ~ 240-nm pitch) (Ishak et al. 2021). Some experiments also have demonstrated that the deflection of nanopillars can increase bactericidal effects (Ivanova et al. 2020; Linklater et al. 2018; Higgins et al. 2020). The deflection of the nanopillars is enhanced by factors such as height, aspect ratio, and bending stiffness.

### Effect of surface wettability

The relationship between surface wettability and bacterial death on nanopatterned surfaces is still inconclusive. When hydrophobic bacteria such as Streptococcus approach the surface, their hydrophobic nature facilitates the removal of interfacial water, leading to the formation of acid–base attractive forces (Olsson et al. 2010). According to the study by Nakade et al., adhered cells increased with hydrophobicity in both flat and nanostructured surfaces, resulting in a higher percentage of bacterial death on highly hydrophobic surfaces (Nakade et al. 2018). It is possible to suggest that hydrophobic surfaces facilitate bacterial attachment by effectively removing interfacial water compared to hydrophilic surfaces, leading to stronger surface adhesions. In addition, hydrophobic surfaces hinder bacterial access to the surface by reducing water retention.

A series of interesting observations was presented by the work of Valiei et al., giving new perspectives on the mechanobactericidal nanopillars (Valiei et al. 2020). Two nanopatterns constructed from NanoSi and NanoZnO showed no bactericidal effect, even though similar structures were claimed to be bactericidal. Thoroughly exploring different experimental reasons, they claim that trapped air bubbles increase the bactericidal effect near the air–liquid interface, whereas the outside exhibited considerable viable cells. The experimental work demonstrated that the liquid–air interface or surface tension could provide external force to induce bacterial death, indicating that normal forces adhering the cell to the nanopillars may not be sufficient. Furthermore, they showed that bacterial death did not occur in regions with low stress (0.17 MPa), but it did occur in regions with larger stress (0.4 MPa).

### Interpreting mechanobactericidal effects through energy-based models

Based on the bactericidal properties of cicada wings, a biophysical model was proposed to explain bactericidal activity and bacterial cell interactions with nanopatterned cicada wings (Pogodin et al. 2013). The model emphasizes that the cell rupture predominantly occurs in the suspended region due to stretching of the cell membrane, without any penetration by the pillars. The experiment also demonstrates the varying resistance of different bacteria, such as *B. subtilis*, *Plancoccus maritimus*, *S. aureus*, and *Pseudomonas aeruginosa*, to the bactericidal effects of cicada wings, highlighting the role of cell membrane strength. However, the study lacked sufficient evidence to support the claim that cell rupture occurs in the suspended region. The analysis within this study adopts a macroscopic perspective, considering the trade-off between the energy gain process of adhesion and the energy loss process of stretching/compression, with the aim of reaching its energy-minimized state compared to the initial state. The model employs a parameter called “stretching degrees” to quantitatively measure the energy required in stretching or compression. Later, Xue et al. used stretching theory to further investigate the physical interactions discussed in models such as the biophysical model. Their investigation revealed that these physical interactions alone are not sufficient to create complete adhesion, and nonspecific forces such as gravitational force and van der Waals forces are necessary to induce adhesion capable of causing cell rupture (Xue et al. 2015). However, the aforementioned forces, such as gravity, are considered negligible, with magnitudes on the order of 10^−15^ N. An Atomic Force Microscopy (AFM) experiment indicated that a force exceeding 2 × 10^−8^N is required to rupture a bacterial cell (Valle et al. 2020). The theoretical model developed by Li et al. incorporates the addition of bending energy to stretching energy, which contributes to the energy loss component (Li 2016). In this model, the total energy change of the system is derived by considering the energy associated with the stretching and area change of the cell membrane, the membrane bending energy, and the contact adhesion energy. In this model, cell membrane deformation is also represented as “stretching degrees.” Their investigation demonstrated that by replicating the geometric attributes of cicada wings, the stretching degrees of the membrane increased by 31% in contrast to a flat surface. They claim that the ability of cicada wings to induce higher stretching degrees in the membrane is a key factor contributing to their bactericidal efficacy. Later, the model was extended by separately treating the area of the top region, the contact adhesion region, and the region immediately near the contact adhesion (Li and Chen 2016). Conclusions were drawn that indicate nanopillars with a large radius and small spacing show enhanced bacterial adhesion to the surface, while nanopillars with a radius smaller than a critical value exhibit suppression in adhesion (Li and Chen 2016). Another model, based on interfacial energy gradient, was proposed, which claims that the interfacial energy gradient is the driving force that propels bacteria into the interior of nanostructures, exerting pressure on the cell wall (Liu et al. 2019). According to this model, when the induced pressure exceeds the critical elastic stress, it causes the cell wall to undergo creep deformation, ultimately resulting in its penetration and cell death. Additionally, the authors demonstrate that wettable substrate materials exert higher pressure on adhered cells due to the smaller contact angle.

A test conducted on a single cell which was placed attached to cicada wing surface, took 3 min and 200 nm depth of travelling for cell rupture to occur, using AFM, applying constant force overtime (Ivanova et al. 2012). Based on this study, it is evident that cell deformation must be extremely high for instantaneous cell rupture to occur. Another study that used AFM to nano indent *E. coli* cells has shown that it requires 20 nN to induce critical cell damage (Valle et al. 2020). So, there is a possibility that deformation of extended period can cause creep deformation, which eventually rupture and lead cell death, as previous model have predicted (Liu et al. 2019). Watson et al. also introduced a simplistic model accounting for the alteration in surface energy and the requisite work for cell bending. This model aligns with the other proposed models, suggesting that when the tensile stress surpasses the strength of the cell, it ruptures, leading to cell death (Watson et al. 2019).

Overall, all these models were derived based on the bacteria reaching an energy-minimized state, compensating for the energy gained from deformation of the bacteria (stretching and bending) through the energy losses due to absorption. According to the models, the stress created by the deformation causes structural damage and cell rupture, ultimately leading to the death of bacteria on the surface. Additionally, the absorption energy due to nano features, and the absorption mechanism is considerable to the extent that it can cause self-destructive deformation either over a short period or an extended time while bacteria adhere to the surface. As indicated previously, additional forces are required to reach the irreversible energy-minimized attachment of the bacteria to the surface. The driving force behind bacteria reaching the energy-minimized state can be the force created by bacterial appendages or kinetic energy in their movements.

### Surface adhesion and motility

Hizal et al. conducted experiments on nanopillared Si surfaces, revealing that the bacterial extracellular polymeric substance (EPS) layer enhances surface adhesion, thereby increasing the effectiveness of killing on nanopatterned surfaces (Hizal et al. 2016). Bandara et al. proposed a similar theory for bacterial death on cicada wings, attributing it to the shear forces generated when immobilized bacteria attempt to move on the nanostructure (Bandara et al. 2017). The authors claim that bacteria firmly attached to the nanopillar through the bacterial EPS layer, and the shear forces created by the movement of the bacteria disrupt the integrity of the bacterial cells, leading to their death. Those conclusions were drawn based on the observation of micrographic images, and the prediction of movement-induced shear failure was made by considering the nature of nanopillar bending. However, later experiments conducted on black silicon nanopillar surfaces elucidated that the bactericidal effect occurs independently of the presence of EPS (Linklater et al. 2017b). This conclusion was derived from the observation that bacterial death occurred within a short timeframe, insufficient for the secretion of EPS (Linklater et al. 2017b). An additional intriguing discovery of the study was the increased bacterial adhesion to the hydrophobic nanopillar pattern compared to the hydrophilic counterpart. Additionally, the percentage of dead cells with respect to attached cells became independent of the surface wettability. The number of bacteria attached to the hydrophobic surface was also considerably high, which agrees with the prior research findings and conclusions (Mitik-Dineva et al. 2009). Based on the results, it is not possible to fully conclude that the killing mechanism is independent of the cellular affinity for a surface, as claimed by the authors of the research work. It is possible that even the attractive forces between hydrophilic surface material and bacteria are sufficient to facilitate surface bactericidal effects for the given surface topology. In the same study, the motility of bacteria as a cause of bactericidal effect was ruled out, as the nanopatterned surfaces exhibited similar killing efficiencies against *P. aeruginosa* and *S. aureus* bacterial cells. Specifically, *S. aureus* was found to be non-motile (Samad et al. 2017). Jindal et al. conducted a study on the effect of bacterial motility on cell adherence and damage on nanostructured surfaces using genetically modified *E. coli* strains, including strains with no flagella, non-motile flagella, flagella with deficient chemotaxis (Jindai et al. 2020). Their findings revealed that motility not only increased the adhesion of bacteria to nano surfaces but also played a vital role in bacterial death on such surfaces. This was demonstrated by the active bacterial percentage being nearly 100% for non-motile strains, while the active cell percentage was 40% after 25 min. These results indicate that the movement of bacteria plays a critical role in bacterial death on nano surfaces. However, for longer durations, all types of bacterial stains including non-motile ones were killed by the nanopatterns, and the authors suggest that the impact of gravitational force might contribute to bacterial death. But prior research has shown that gravitational force was negligible. This provides clear evidence that cell movement could lead to an increased rate of bactericidal death. However, their results suggest that this increase is primarily due to the greater number of surface adhesions in motile strains rather than a direct effect of their movement. Motile strains are clearly capable of sensing and reaching surfaces easily, which is part of their surface adhesion mechanism, as explained in the above section. Consequently, in the results, fewer cells were attached, even for motile strains that cannot detect the surface (strains with deficient chemotaxis). In the absence of sensory and motility capabilities, bacteria take a longer period of time to reach the surface. This could explain why those strains took considerably longer to be killed by the nanopatterned surfaces. Ishak et al. have illustrated the importance of surface proteins in the bactericidal mechanism of nanostructured surfaces by using trypsinized *E. coli* bacteria (Ishak et al. 2021). Trypsinization removed surface proteins that enable cells to adhere to surfaces. Trypsinized bacteria showed less vitality, while untreated bacteria showed high vitality when in contact with a nanostructured surface.

### Programmed cell death (PCD) in bacteria

Programmed cell death (PCD) is a genetic process of cellular suicide that can be triggered by various factors. It is an evolutionary adaptation where cell sacrifice sustains the survival of the remaining cells in the colony (Allocati et al. 2015). PCD follows a controlled sequence of signals involving specific molecules. In contrast, non-programmed cell death, also known as accidental cell death, is an uncontrolled biological process.

Apoptosis is also a form of programmed cell death (PCD) where cell death occurs following particular molecular steps in order to eliminate defective cells (Metzstein et al. 1998). Autolysis belongs to programmed cell death and is responsible for eradicating severely damaged cells (Zhang et al. 2022; Lewis 2000). In the autolyzing process, self-digestion of cell wall occurs due to peptidoglycan hydrolases called autolysins (Shockman et al. 1996). Research has demonstrated that stress-induced accumulation of reactive oxygen species (ROS), which can be toxic to cells, leads to types of PCD, which can be either apoptosis or controlled necrosis (Foti et al. 2012; Wang and Zhao 2009; Dorsey-Oresto et al. 2013; An et al. 2024). The ROS primarily include hydroxyl radicals, hydrogen peroxide, and superoxide anions. It has been shown that ROS can contribute to cell death even after the stressor that triggered their accumulation is removed (Hong et al. 2019). However, for this self-driven death process to occur, the ROS threshold must exceed a critical level, and if this condition is met, the cell will be destroyed even if the damage caused by the initial stress is not sufficient to directly kill the bacteria (Hong et al. 2019). Therefore, it is possible that ROS can lead to cell death if the stress created by the nanostructures is sufficient to generate ROS that exceed the required threshold.

**Fig. 6:**
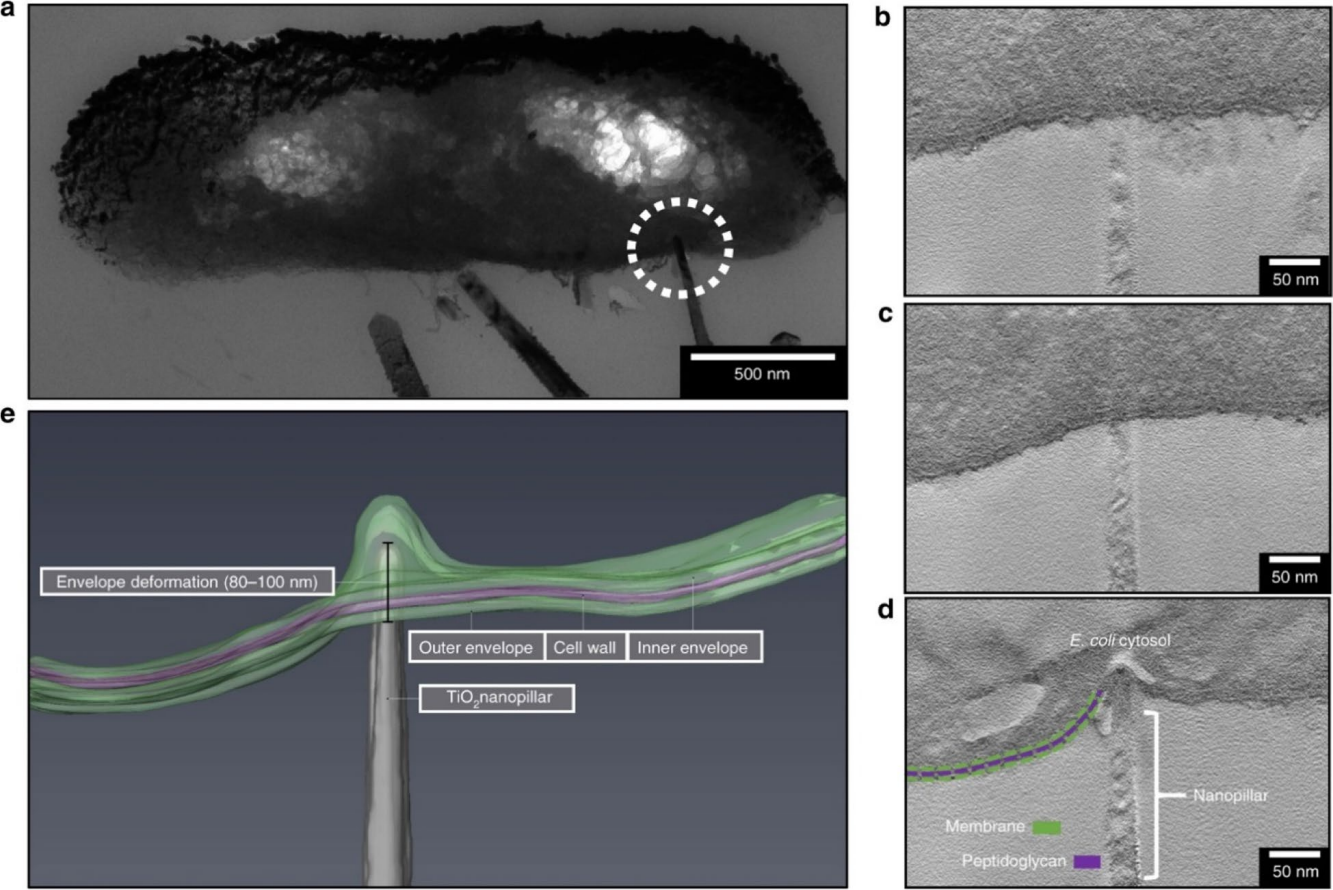
**a** Possible penetration of *E.coli* envelope (dashed white circle) by single nanopillar. Analysis of tomographic slices from the **b** start, **c** middle, **d** and point of nanopillar contact, indicating the envelop indentation without penetration. **e** 3D reconstruction of electron tomography was performed on *E. coli* cells interacting with TiO_2_ nanopillars (reprinted with permission from ref (Jenkins et al. 2020) Copyright 2020 Nature Publications)

Liu et al. have illustrated that graphene-based materials can disrupt bacteria cells through membrane damage as well as oxidative stress (Liu et al. 2011). Jenkins et al. have confirmed in their studies that some degree of mechanical deformation and penetration causes bacteria death on nanopatterned surfaces, using experiments conducted on TiO_2_ nanopillared surfaces by observing images (see Fig. 6) obtained through transmission electron microscopy (TEM) and focused ion beam SEM (FIB-SEM) (Jenkins et al. 2020). Their research also highlights that bacterial death can occur without cell rupture through PCD because of the oxidative stress induced by the deformations, which are revealed by their proteomic analyses. Proteomic analysis has revealed a substantial concentration of differentially expressed proteins linked to oxidative stress. This phenomenon of ROS-induced PCD is found to be self-amplifying, as supported by prior research (Hong et al. 2019).

Zhao et al. observed ROS-induced apoptosis-like PCD in the Gram-negative bacterium *Pseudomonas aeruginosa* on a black silicon nanostructured surface. Their research has shown that mechanical stress created by the nanostructures leads to PCD not only in adhered cells but also in sub-lethally physically damaged cells even after being transferred to a non-stressed environment (Zhao et al. 2022). In most research, it is evident that substantial concentrations of ROS are present, generated in response to the mechanical stress acting on the cell membrane. However, further studies are required to determine the concentration of these species sufficient to trigger PCD.

Daimon et al. investigated the death of *E. coli* cells and liposomes on Au nanostructure arrays (Daimon et al. 2023). Their studies have revealed damage only to *E. coli* cells, despite both cell types being attached to the structures and having similar mechanical strength in their outer membranes. Since liposomes are pseudo-cells composed only of a lipid bilayer, the authors suggested that bacterial death on nanostructured surfaces is mainly caused by an autolytic mechanism. However, in this study, it is difficult to determine whether adhered pseudo-cells exert similar pressure on the cell membranes as *E. coli* cells do, due to their different shapes and lack of appendages. Mimura et al. have revealed important findings related to autolytic type PCD (Mimura et al. 2022). They conducted tests on four variants, three of which lack certain enzymes that facilitate the PCD. In the study, the *E. coli* strain lacking the Slt70 (soluble lytic transglycosylases) enzyme showed very little cell death compared to the wild-type *E. coli* with all autolytic enzymes. Additionally, *E. coli* strains lacking MltA (membrane-bound lytic transglycosylase A) and MltB (membrane-bound lytic transglycosylase B) autolytic enzymes. In *E. coli*, Slt70 is located in the periplasm, while MltA and MltB are located on the outer membrane (Lewis 2000). These enzymes were prominent during cell division and are major components of the autolytic system in *E. coli* (Höltje 1995). As the authors stated, the Slt70 enzyme is critical for the cell’s autolytic process, and cell lysis originates from the inner part of the cell through an enzymatic reaction. Another important result found in the same research was that adding Mg2 +, which is an autolysis inhibitor, to the process to the process stopped *E. coli* cell death on nanostructures, which had previously killed the bacteria efficiently. This suggests that the cell death mechanism is an autolysis process that is triggered by the cell lysis enzymes. According to the research, it is evident that stress created by the nanostructures has triggered the cell autolysis mechanism, which eventually leads to cell death.

## Conclusion

### Summary and conclusions: mechanobactericidal mechanism

Bacterial death on nanostructured surfaces may seem like a simple process from the surface level, but the mechanism behind it is complex and requires a series of thorough investigations to establish an acceptable understanding. The bactericidal process begins with bacteria sensing the surface and attaching to it. Mechanosensing plays a vital role in enabling bacteria to reach and efficiently attach to the surface. Although motility facilitates speed that reach to the surface, non-motile bacteria can still reach nanosurfaces through other mechanisms, albeit taking longer. According to studies employing the extended DLVO theory, energy-based models, and finite element analysis, the bacterial adhesion process is critical for developing substantial stress in the bacterial membranes. According to various hypotheses related to the bactericidal mechanism on nanostructures, we can mainly categorize them into two parts. One is that cell death is solely caused by stress-induced mechanical failure of the cell membrane. These include penetration and substantial membrane fracture, creep failure after prolonged exposure to stress, and shear fracture due to bacteria attempting to move. There is sufficient evidence to suggest that movement-induced shear failure may not be the cause of bactericidal activity on nanostructures. However, if the stress exceeds a certain value, it is possible to severely damage the cell through penetration or fracturing, killing the bacteria. But is this the only mechanism that could cause cell death in nanostructures? Based on our investigation, it is clear that even if the stress levels are not high enough to substantially damage the cell envelope and cause mechanical failure, other forms of cell death, such as PCD (programmed cell death), could still occur, identifying the cell as defective or damaged even after the initial stress precursor is removed. In the case of PCD-based cell death, ROS (reactive oxygen species)-induced cell death is one hypothesis, but it requires further evidence to conclude whether enough ROS are generated to activate PCD. A controlled form of necrosis, specifically autolysis, can also lead to cell death. This process is enzyme-driven, and research has shown conclusive evidence to show that cell death is caused by the autolytic process in nanostructures as a response to damaged cell envelopes.

### Future direction

The mechanobactericidal effect is advantageous in many ways, as it is entirely dependent on surface topography and does not involve antibiotics or other chemicals. However, if the features can be scaled up to the microscale, they would offer additional benefits, such as enabling easy integration into various components like door handles. A downside of the nanofeatures is that slight pressure could easily damage the surface nanofeatures, reducing their effectiveness. Therefore, understanding the mechanisms of cell death is crucial. The topology needs to be specifically developed to target the programmed cell death (PCD) mechanism triggered by stress induced by the mechanical structures. In addition to autolysis, ROS-induced PCD also needs to be studied in depth to determine which structures could generate sufficient ROS to activate PCD. This approach will also enable the development of highly efficient, self-amplifying, and self-driven bactericidal surfaces cost-effectively.

## Data Availability

No datasets were generated or analysed during the current study.
